# Development of a fruit size estimation method using Mask RCNN for water stress estimation in Satsuma mandarin trees

**DOI:** 10.1371/journal.pone.0324278

**Published:** 2025-07-11

**Authors:** Ringo Komiya, Mitsunori Iwasaki, Harshana Habaragamuwa, Hiroshi Fukamachi

**Affiliations:** 1 Institute of Fruit Tree and Tea Science, National Agriculture and Food Research Organization, Shizuoka, Japan; 2 Core Technology Research Headquarters, National Agriculture and Food Research Organization, Tsukuba, Ibaraki, Japan; Public Library of Science, UNITED KINGDOM OF GREAT BRITAIN AND NORTHERN IRELAND

## Abstract

Satsuma mandarins can produce high-sugar fruits through effective water stress management during the summer and fall seasons. Estimating water stress is crucial as it allows for precise irrigation management, directly impacting fruit quality and yield. In recent years, the development of automatic irrigation technology has increased the demand for methods to obtain digital data on water stress and related data. This study presents a smartphone-based fruit size estimation method that leverages the correlation between fruit growth and tree water stress. The developed method combined Mask RCNN and a regression model. The coefficients of determination between actual and estimated fruit diameters, between fruit growth and water stress, and between measured diameter and water stress were 0.988, 0.7789, and 0.7349, respectively. This study’s method of accurately estimating fruit diameter using smartphone technology can be integrated with irrigation systems to optimize water usage based on real-time assessments of fruit development and water stress.

## Introduction

Satsuma Mandarin (*Citrus unshiu* Marcov.) is the most widely planted fruit tree in Japan [1], and consumer demand for fruits with high-sugar content and appropriate acidity is high. Optimal water stress (drought stress) during the summer and fall seasons increases fruit sugar content [2,3]. Cultivation methods, such as mulch cultivation [4,5], shielding-mulch cultivation [6], and root zone restriction cultivation [7], can provide water stress in the open field environment in Japan. However, the defoliation observed in trees subjected to strong water stress [8] indicates that excessive water stress can damage trees. Therefore, the stable production of high-sugar fruits necessitates continuously investigating water stress and determining whether and to what extent irrigation is necessary based on this information.

An effective method for assessing tree water stress involves the pressure chamber-based measurement of the pre-dawn leaf water potential, Ψ_PLWP_ [9]. However, farmers often do not use this method because it requires pre-dawn work and expensive equipment, so a simpler water stress estimation method is needed. A simple method for estimating water stress involves measuring the fruit enlargement rate. This method is based on the reported correlation between the fruit enlargement rate and water stress degree [8]. Kaibara and Shindo [10] reported that when the fruit daily growth rate is less than 0.25 mm, it is an indication that Ψ_PLWP_ of −0.8 MPa or less is being applied. Notably, farmers regularly measure the fruit lateral diameter using Vernier calipers. Nevertheless, this method is somewhat complicated because the fruit’s equatorial plane is not a perfect circle, so measurement requires skill, and the amount of fruit enlargement must be calculated manually from the diameter data. Furthermore, recent advancements have introduced irrigation facilities equipped with Internet communication capabilities, highlighting the need for a data acquisition method that can easily integrate with these systems. Linking water stress estimation methods with irrigation facilities can facilitate automated and straightforward irrigation decisions, easing the workload for farmers and consistently producing high-quality fruits yearly.

Our aim was to develop a cost-effective measurement technique using a digital device that correlates with water stress as effectively as, or better than, the conventional method (Vernier calipers). Notably, digital device-based fruit size estimation methods for white mulch-lined mandarin fields remain lacking.

One approach to obtaining digital data on fruit size involves utilizing a smartphone to capture fruit images. The smartphone ownership rate per household in Japan, which exceeds 90% [11], suggests that many farmers could inexpensively adopt technology utilizing smartphone cameras. Murali and Won [12] estimated orange diameter using a color camera and an ultrasonic sensor. Wang et al. [13] used a color camera and three distance estimation methods to estimate mango size. Stajnko et al. [14] employed thermal images to estimate apple size. Wang et al. [15] demonstrated the use of a monocular camera by placing a mango on a piece of paper printed with size reference marks, photographing it, and then estimating the mango’s size from the image processing results.

Fruits can be challenging to detect using conventional methods, such as binarization. In such cases, deploying deep learning techniques may solve this problem. Koirala et al. [16] explained that traditional algorithms might be ineffective in complex settings and reported that a deep learning model was robust when used with images from different orchards, cultivars, and lighting conditions in mango detection. Other studies using deep learning have been reported. For instance, Apolo et al. [17] used deep learning to estimate the size of oranges from UAV-acquired images. Ferrer et al. [18] estimated apple size from RGB-D images using a multitasking deep neural network. Although these studies also provided good size estimates, they were optimized for the specific devices used in each study and would be difficult to adapt to use with a smartphone monocular camera.

Mask RCNN [19] is a widely used instance segmentation method with an excellent code base [20]. As examples of its use in plants, Wang et al. [21] used it for tea bud and leaf recognition and reported high detection rates. Lopez et al. [22] utilized it to detect bell pepper fruit in greenhouses and reported good results. Based on these reports, Mask RCNN was adopted as our architecture because we needed to accurately estimate the fruit diameter, and Mask RCNN can offer accurate segmentations [21]. Since Mask RCNN image processing can output the area of an object, we considered that this value could be input into a regression model to achieve a more accurate estimation of fruit diameter.

This study presents a fruit diameter estimation method developed using Mask RCNN and a regression model to estimate leaf water potential from smartphone images. The relationship between the estimated and actual fruit diameters was determined, as well as the relationship between the estimated fruit diameter and leaf water potential.

## Materials and methods

### Field experimental site and plant material

Plant materials were fruits belonging to adult—13th grade as of 2022—“Okitsu-wase” Satsuma mandarin trees grafted on trifoliate orange (*Poncirus trifoliata* L. Raf.) rootstock growing in a field at the NARO Institute of Fruit Tree Science in Shimizu, Shizuoka, Japan. Since this field was under our control, no use permit was required. The trees were planted on ridges running in a north-south direction on gentle sloping terrain (2:100). The soil was clayey and had high water retention capacity. Inter-row spacing was 4.6 m, with the trees planted 2 m apart within the rows. Trees were grown in either non-mulch, mulch, or shielding-mulch cultivation. In 2022 and 2023, 21 healthy trees with an average fruit bearing were used for a drought stress experiment. Table 1 shows the number of trees for each cultivation method used in each year. We didn’t use cultivation method as treatment, just increase diversity of water stress.

**Table 1:** Number of Trees for Each Cultivation Method Used in Each Year.

	Non-mulch cultivation	Mulch cultivation	Shielding-mulch cultivation
2022 (training/validation data)	0	5	19
2023 (test data)	3	8	11

Mulch cultivation is a cultivation method in Japan, where rainfall is frequent, in which mulch sheets are deployed on the ground surface to prevent rainwater from entering the root zone and to dry out the soil in order to provide water stress to the tree. Shielding mulch cultivation is a method in which, in addition to mulch sheets, water-barrier sheets are installed on the sides of the rows to prevent rainwater from entering the pathways and to provide stronger water stress. Trees in all cultivation methods were equipped with watering tubes (Mist ace saiteki 04L-03, Sumika Agrotech Co.,Ltd., Japan) to irrigate in case of excessive water stress.

### Drought stress experiment on citrus trees

To provide water stress to trees under mulch and shielding-mulch cultivation, mulch sheets (Tyvek760AG, DuPont-Asahi Flash Spun Products Co., Japan) were deployed from late June to middle November (harvest time).

As a water stress indicator, the pre-dawn leaf water potential (Ψ_PLWP_) was measured for between 0300 and 0500 h (Local Time: GMT + 9 h) before sunrise using a Scholander-type pressure chamber (Model 600 Pressure Chamber Instrument, PMS instrument, Corvallis, OR) (9). Two leaves near the tip of the spring branch per tree per measurement were used for each measurement, and the average value was used as the measurement value. Measurements of the trees were taken every 7–10 days on clear days.

**Fig 1:**
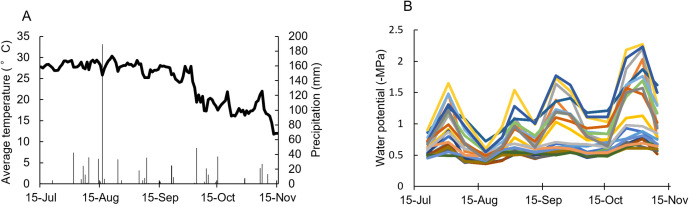
Weather conditions and water stress of each tree in the test dataset (2023). **(A)** Average temperature (line graph) and precipitation (bar graph). **(B)** Water stress of the trees used in the experiment (n = 22).

Fig 1 presents the weather data for the field and the water stress of each tree in the test dataset (2023). Generally, we were able to collect from trees with diverse patterns of water stress conditions, ranging from nearly non water-stressed trees with Ψ_PLWP_ hovering around −0.5 MPa to trees with Ψ_PLWP_ as high as −1.5 MPa or lower, but all trees were under low water stress in mid-August due to heavy rainfall caused by a typhoon on August 16, which flooded the fields. Since the trees were not subjected to water stress at mortality levels (Ψ_PLWP_ below −2.0 MPa) from the beginning of the survey to mid-October, irrigation was not performed.

### Installation of 2D bar codes

**Fig 2:**
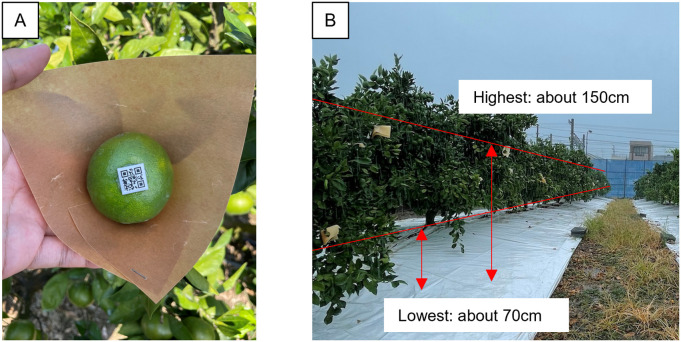
Adhesion treatment of size object to fruit and status of attachment of treated fruit. **(A)** Fruits processed by gluing objects of size. **(B)** Fruit set of treated fruit.

To estimate fruit size with a monocular camera, we installed a plastic piece (printed 2D bar code for unique fruit ID, 2 mm thick, 15 mm per side) on the fruit’s top using silicone adhesive (TSE382-C, Momentive Performance Materials, USA) in late July. Fig 2 shows the treated fruits and their fruit sets. We treated 7 fruits per tree in 2022 and 5 fruits per tree in 2023. Fruits to be treated were randomly selected from those of average size in the tree as of late July, before water stress was applied, at a height (70–150 cm above ground level) that was easy to photograph. If a plastic piece was dislodged from the fruit, it was glued to the same fruit again. To facilitate the isolation of fruit regions from the captured fruit images, umbrella hanging was performed using water-resistant paper 15 cm square from the fruit stalk.

### Collection of fruit images and fruit diameter data

**Fig 3:**
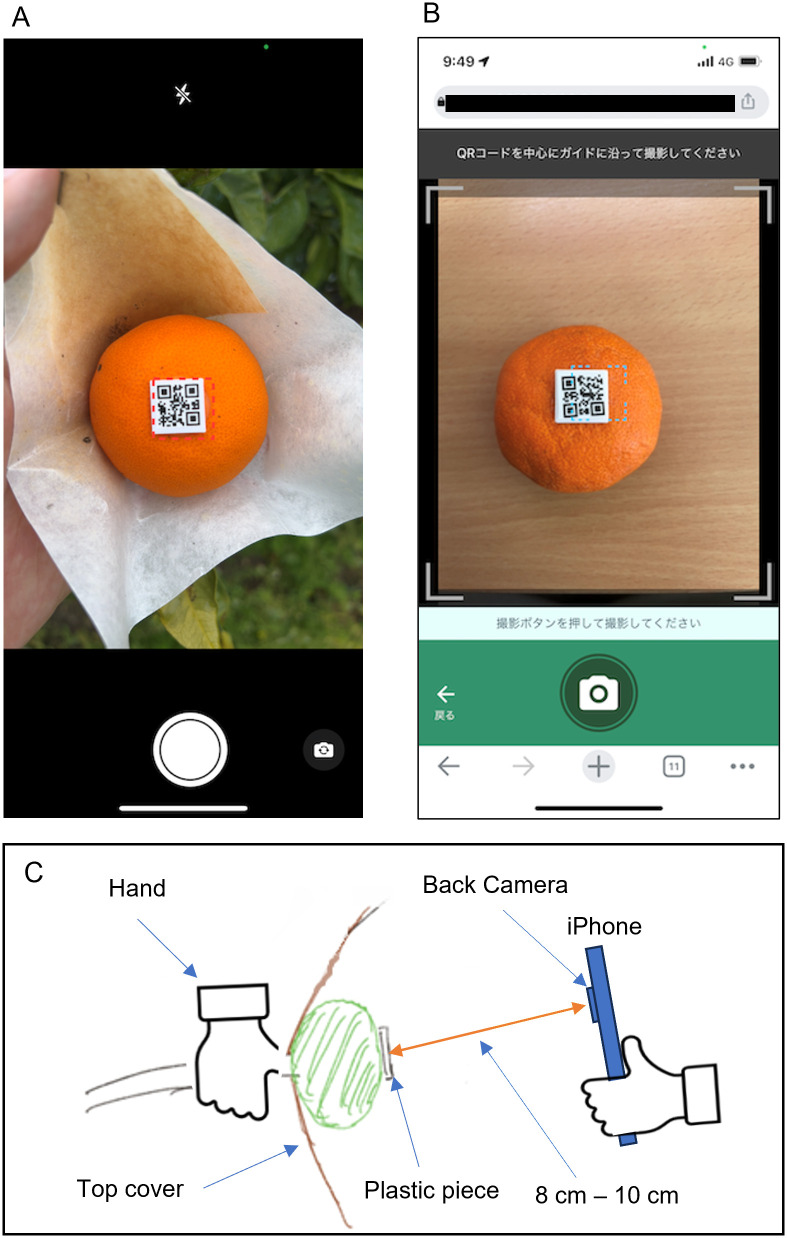
Shooting screen developed for capturing fruit images. (A) Shooting screen for high-quality images in 2022 and 2023. (B) Shooting screen for low-quality images in 2023.

The 2022 and 2023 fruit images were taken using an iPhone 12 Pro wide-angle camera (Apple Inc., USA). To align the conditions of the images to some extent, an application was created that implemented a frame as a sighting guide for a plastic piece on the shooting screen (Fig 3). Using these applications, images were taken.

In 2022, a high-resolution image capturing function (3024 × 4032 pixels) was developed as an iOS application for the purpose of more accurate data acquisition, and 17901 images were collected. In 2023, a low-quality image shooting function (480*640 pixels) with low data volume was developed separately as a web application to send images to a server for analysis as a web service, and 9693 and 9192 high-resolution and low-resolution images were collected respectively. Shooting was done without precipitation and under a variety of conditions ranging from overcast to clear skies.

The photos were taken between 6:00 a.m. and 9:00 a.m., coinciding with the day of the water stress measurement. In our preliminary testing we examined different methods of photographing different conditions. We found that if the camera is too close to the fruit, it cannot capture the outline of the larger fruit, but if it is too far away, multiple fruits are reflected in the image, making it difficult to tell which fruit is the focus of the camera. Based on these verifications, we established the following criteria: 8–10 cm from the fruit and 0–20 degrees from the fruit, as the conditions in which the camera can capture the outline of the fruit at the time of harvest and is somewhat close to the fruit. In this study, all fruit images were taken using this criterion, and we confirmed that the largest size fruit contours could be captured. Within the same shooting day, multiple images—about 7 high-quality images per processed fruit in 2022, and about 5 each of high- and low-quality images in 2023—were taken under the aforementioned conditions. Two diameter points per fruit were obtained for each fruit using a digital caliper during the same period as the image acquisition. This allows the fruit diameter data associated with all captured images to be aligned.

### Fine-tuning of Mask RCNN

To detect and segment fruit and plastic pieces, we used the Mask RCNN method (19). For retraining the Mask RCNN model, 550 images—from the 2019 preliminary exam and some from the 2022 exam—were annotated using the VGG Image Annotator [23]. The backbone of the model was Resnet-101, pretrained by the coco dataset. In retraining, the settings were changed from a model that recognized 91 categories to a model that recognized fruits and plastic pieces. 500 epoch retraining was performed using annotated data split training: validation = 7:3, and the weight with the lowest validation loss was used for image analysis. For image analysis, the acquired images were input to Mask RCNN, and the areas of fruit and plastic piece regions were obtained from the output data. Since the output of the Mask RCNN is the data in the middle of the fruit diameter estimation pipeline, we did not create a test dataset for the Mask RCNN.

### Creating regression equations

As a method for estimating fruit diameter using the output of the Mask RCNN, the area ratio of fruit to plastic pieces was calculated using 2022 data, and a regression equation of fruit diameter was developed. The regression equation created was applied to the output values from the Mask RCNN for the 2023 image to obtain the estimated fruit diameter. Equations were created and validated for each of the two resolution image patterns (3024 × 4032 and 480 × 640 pixels). To create an equation for low resolution from the 2022 data, a separate dataset of 2022 images converted to low resolution was created.

To verify the accuracy of the estimated fruit diameter obtained, the coefficient of determination, mean absolute error (MAE), and root mean square error (RMSE) of the estimated and measured fruit diameters were calculated for the 2023 data prepared for testing. SciPy’s function [24] was used to calculate the coefficient of determination, while the MAE and RMSE were calculated by implementing the following equations based on NumPy’s function [25]:


$$\begin{array}{c} MAE=\frac{1}{n}\sum_{i=1}^{n}\left|D_{ei}-D_{GTi}\right|, \end{array}$$ (1)

$$\begin{array}{c} RMSE=\sqrt{\frac{1}{n}\sum_{i=1}^{n}{\left(D_{ei}-D_{GTi}\right)}^{2}}. \end{array}$$ (2)

where n is the number of observations, D_e_ is the diameter estimation, and D_GT_ is the diameter ground truth.

### Regression models

To develop a fruit diameter estimation method that is more accurate than the method incorporating a Mask RCNN-obtained area ratio, we tested a regression model using the output values from Mask RCNN. To estimate fruit size from the Mask RCNN output, several regression models were created using scikit-learn [26]. We used linear regression, ridge regression, lasso regression, elasticNet regression, random forest, gradient boosted decision trees (GBDT), and support vector regression (SVR) for our performance comparisons.

All models were trained as models predicting fruit diameter (measured by Vernier caliper) with fruit area, plastic piece area, and ratio of fruit area to plastic piece area (obtained by Mask RCNN outputs). All training and validation explanatory variables were normalized based on the 2022 data pattern. Models were created and validated for each of the two resolution image patterns (3024 × 4032 and 480 × 640 pixels), as well as regression equations. Data from the resized 2022 image were used to develop the models for lower resolution. Models were trained on the Mask RCNN results for the 2022 image and tested with the Mask RCNN results for the 2023 image, so the training (training and validation dataset) and testing (testing dataset) were completely separated. Various combinations of parameters used as explanatory variables were prepared, optimized, and verified with test data to determine which parameters are best suited for use as explanatory variables.

To verify the accuracy of the estimated fruit diameter for each model, the coefficient of determination, MAE, and RMSE were calculated using the same means as for the verification of fruit diameter estimation by area ratio.

### Determination of fruit growth by each method and integrated water stress

Based on the caliper-measured actual fruit diameter and the developed method-estimated fruit diameter, the amount of fruit enlargement was calculated for the entire survey period and for each month.

Data on fruit diameter at the beginning and end of the period under study were used to calculate the amount of fruit enlargement. The caliper measurements were taken by averaging two points, and the development method measurements were taken by randomly selecting one of the images taken on the same day. The calculated fruit diameter was converted to an average value per tree.

As an indicator of water stress for the entire period of interest, the following equation was used to calculate integrated water stress (SΨ_x_) with some modifications to Iwasaki’s method (8).


$$\begin{array}{c} S\psi_{x}=\left|\Sigma \left(\frac{\psi_{i}+\psi_{i+1}}{2}-c\right)n\right| \end{array}$$ (3)

In this equation, x is the period of interest, i is the data from the last measurement date before the period, c is the Ψ_PLWP_ in without water stress tree (−0.4 MPa) and n is the number of days between measurements from i to i + 1.

SΨ_x_ was calculated in the same way for the entire measurement period and for each month, and the relationship between each method and the amount of fruit growth was verified.

## Results and discussion

### Image analysis by Mask RCNN

**Fig 4:**
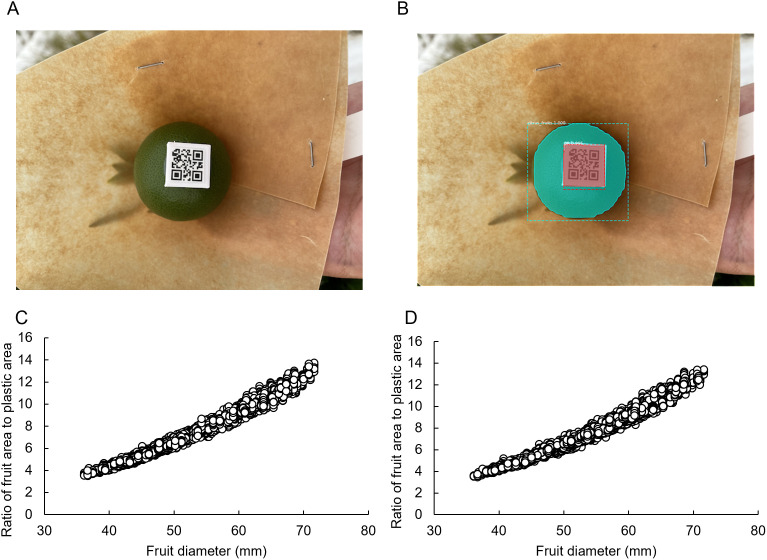
Mask RCNN analysis results and trends. **(A)** Input image. **(B)** Output image. **(C)** Relationship between the area ratio of fruit and plastic pieces and fruit diameter using high-resolution images (n = 9693). **(D)** Relationship between the area ratio of fruit and plastic pieces and fruit diameter using low-resolution images (n = 9192).

To verify the Mask RCNN analysis results, images taken in 2023 were input to the retrained Mask RCNN and its output values were obtained. Fig 4A and 4B show representative detection examples. The Mask RCNN successfully masked the fruit and plastic piece with reasonable accuracy, although some errors remained in the detection results. As a method for evaluating fruit size using Mask RCNN, the area ratio of the fruit to the plastic piece was calculated and compared with the actual diameter measured by calipers (Fig 4C and 4D, Table 2). Significant outliers were not observed (Fig 4C and 4D), and fruit or plastic pieces were detected in all images, suggesting that the retrained Mask RCNN could recognize fruit and plastic pieces with certain accuracy in all images.

**Table 2:** Value of the Evaluation Function Between Prediction Diameter Using the Area Ratio of Fruit and Plastic Pieces from the Output of Mask RCNN and Caliper Measurements.

Evaluation function	high resolution	low resolution
coefficient of determination (R^2^)	0.979	0.977
mean absolute error (MAE)	0.883	0.952
root mean squared error (RMSE)	1.194	1.288

The estimation equation was developed from the area ratio of fruit to plastic pieces and fruit diameter by caliper in 2022 and validated by test data (2023).

The area ratios were calculated based on the Mask RCNN output results for the 2022 image, and the regression equation with the measured fruit diameter was adapted to the 2023 image (see results in Table 1). The estimation results demonstrated high accuracy: the coefficient of determination was 0.979 and 0.977, MAE was 0.883 and 0.952, and RMSE was 1.194 and 1.288 for the high- and low-image quality images, respectively. However, a scatter plot of the estimated and measured values was quadratic, and the error tended to increase as the fruit size increased (S1 Fig). Since the area ratio of fruit to plastic strip is two-dimensional data, whereas fruit diameter is one-dimensional data, a scatter plot of the two would have resulted in a quadratic curve.

**Fig 5:**
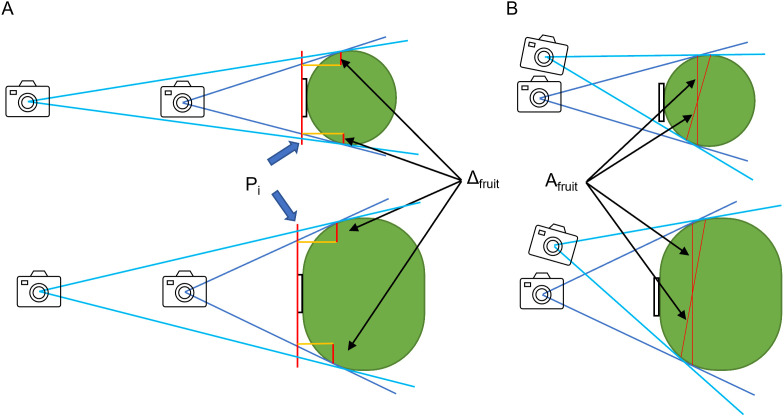
Effect of changing the distance and angle from the plastic piece on the reflection on a monocular camera. **(A)** Effect of changing the distance from the plastic piece on the reflection on a monocular camera. **(B)** Effect of changing the angle from the plastic piece on the reflection on a monocular camera. P_i_ indicates the position of the image with respect to the plastic piece, Δ_fruit_ indicates the fruit area that cannot be evaluated when the image position is set to P_i_, and A_fruit_ indicates the area of fruit for each camera position.

**Fig 6:**
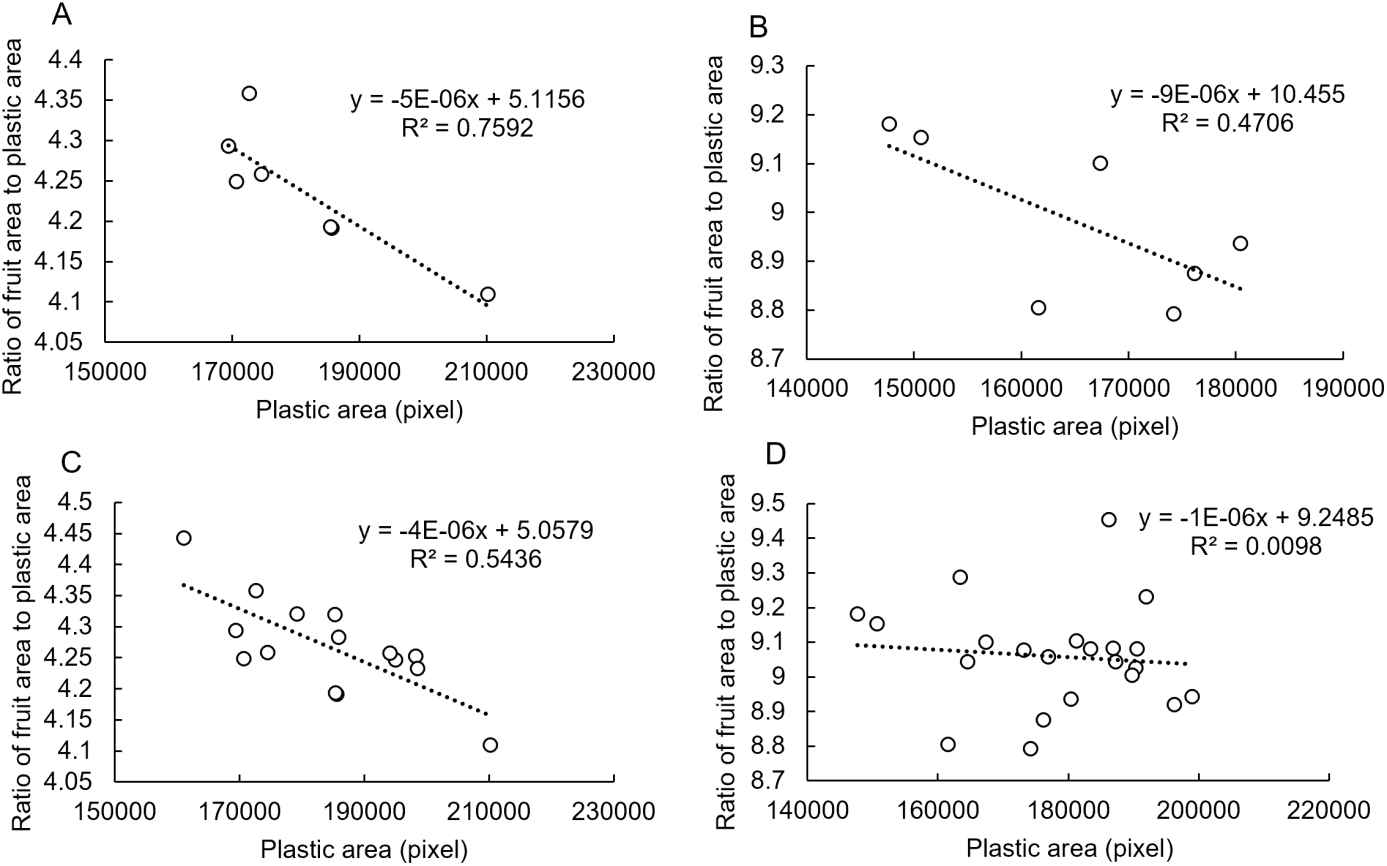
Relationship between area ratio and plastic piece area. (A) 1 fruit 40 mm in diameter. (B) 1 fruit 60 mm in diameter. (C) 2 fruits 40 mm in diameter. (D) 3 fruits 60 mm in diameter.

To understand how this propensity occurs, we considered the effect of the camera position (distance and angle to the fruit) on the image acquired by the monocular camera (Figs 5 and 6). As an effect of the fruit–camera distance, the evaluation position of the photo is fixed at P_i_ when using the plastic piece as a size indicator (Fig 5A). Therefore, although the image captures a position close to the equatorial plane with respect to the fruit, the area of the fruit available for use during evaluation is reduced by Δ_fruit_, which varies with P_i_. The Δ_fruit_ value probably increased with a shorter camera-to-fruit distance and a larger fruit size. To verify this with actual data, we plotted the area of the plastic piece versus the area of the fruit and the plastic piece for a fruit 40 mm and 60 mm in diameter (Fig 6A and 6B). In both graphs, a larger plastic piece corresponded to a smaller area ratio. Additionally, the regression line slope for the plotted data became steeper with increasing fruit size, which is consistent with our discussion.

Before discussing angles, we will discuss the growth of the satsuma mandarin fruit and its shape. In the early growth stages, the fruit is nearly spherical, and its longitudinal section is close to a perfect circle. However, as the fruit grows, its longitudinal section becomes more like a hyper ellipse. Inoue [27] reported that the correlation function between fruit diameter and flatness was 0.461, which translates to a coefficient of determination of 0.21, suggesting that variation in fruit flatness = fruit longitudinal section occurs from fruit to fruit. Fig. 5B illustrates the effect of changing the camera angle on the area of the fruit reflected in the camera. For a small fruit with a longitudinal section close to a perfect circle, the area of the fruit captured by the camera did not change significantly with changes in angle. However, for a large fruit with a hyper-elliptical longitudinal section, the area captured by the camera could change significantly depending on the camera angle. To verify this with actual data, we plotted the area of the plastic piece and the area of fruit and plastic piece in several fruits of 40 mm and 60 mm diameter (see Fig. 6C and 6D). The 40-mm diameter fruit showed some correlation when multiple fruits were plotted, whereas the 60-mm diameter fruit did not show any correlation. This is because the smaller fruits had similar shapes, leading to a consistent correlation. In contrast, the 60-mm diameter fruits exhibited no correlation due to variations in flatness and the divergent effects of camera angles, which aligns with the discussion in the illustration.

These results suggest that although the output of Mask RCNN by itself has a high coefficient of determination, a function to compensate for the distance and angle between the camera and the fruit is necessary for a more accurate estimation.

### Results of regression models

**Fig 7:**
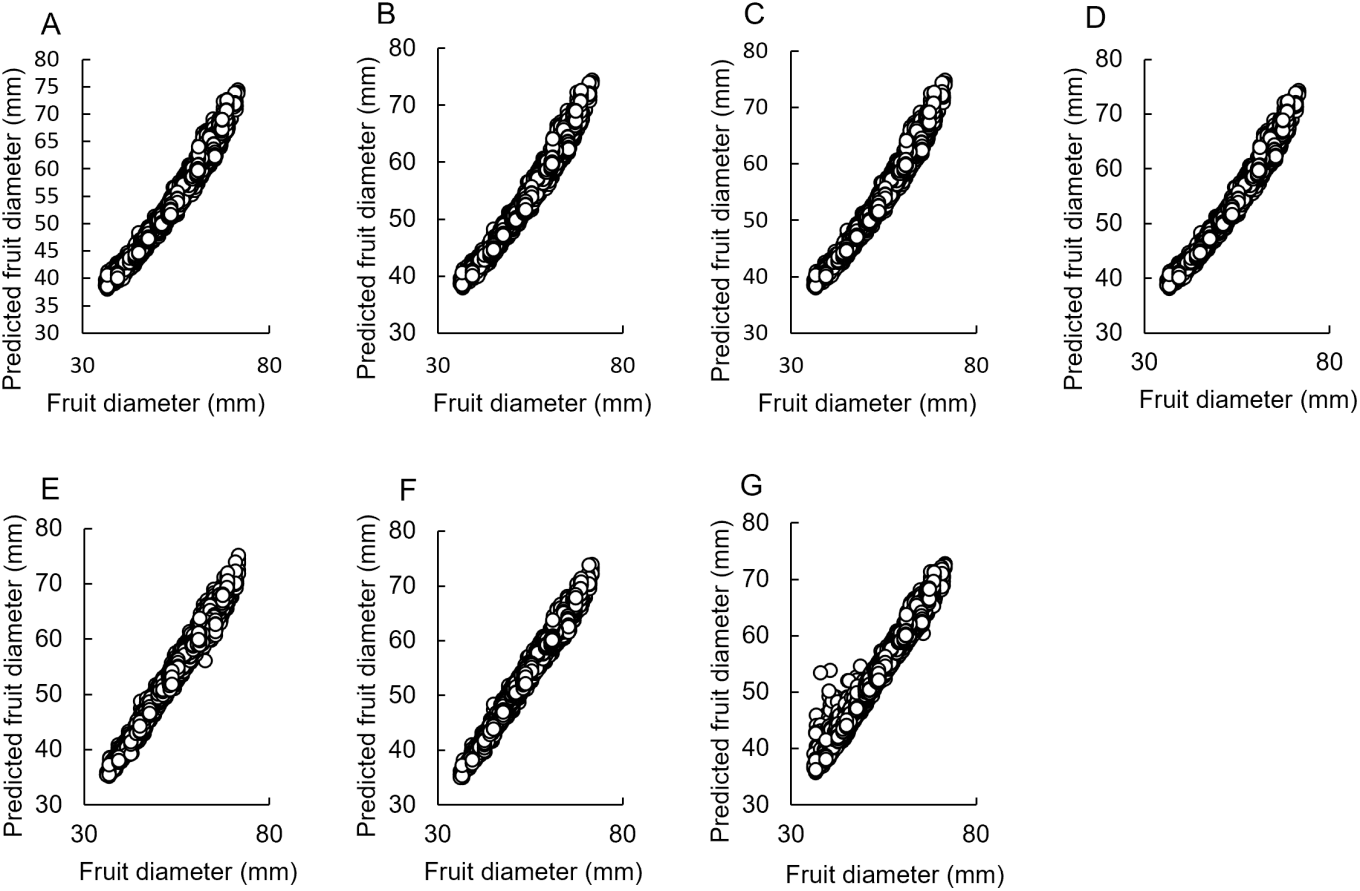
Fruit diameter prediction results for each regression model. **(A)** Linear regression. **(B)** Ridge regression. **(C)** Lasso regression. **(D)** ElasticNet regression. **(E)** Random forest. **(F)** Gradient boosting decision tree. **(G)** Support vector regression. All plot data utilized high-quality image outputs from 2023 (n = 9693).

To estimate the fruit diameter by correcting for errors introduced by the distance and angle between the camera and the fruit, we considered using regression models. Fig 7 depicts the relationship between the estimated and measured values for each model. For both the models for high- and low-quality images, linear regression and its derivatives Ridge regression, Lasso regression, and ElasticNet regression were slightly curvilinear, while the nonlinear models random forest, GBDT, and SVR showed linear regression results. Outliers—data with a difference of 5 mm or more between measured and predicted values—were observed in the SVR and random forest, but not in GBDT.

Table 3 contains the values of the evaluation function for each model. The coefficients of determination were highest for GBDT for the model of high-quality images, and for random forest and GBDT for the model of low-quality images. MAE showed the lowest SVR for both image quality models. MSE and RMSE showed the lowest GBDT values for both image quality models. Based on these results, the SVR demonstrated good performance with MAE but raised concerns about generating outliers. In contrast, GBDT did not produce outliers, making it the best model for this study.

**Table 3:** Value of Each Evaluation Function for Each Regression Model.

	Evaluation function	linear regression	ridge regression	lasso regression	ElasticNet	Random Forest	GBDT	SVR
high resolution	coefficient of determination (R^2^)	0.980	0.980	0.981	0.980	0.985	0.988	0.983
	mean absolute error (MAE)	0.945	0.944	0.928	0.954	0.917	0.857	0.835
	root mean squared error (RMSE)	1.166	1.165	1.147	1.175	1.132	1.045	1.095
low resolution	coefficient of determination (R^2^)	0.976	0.976	0.977	0.976	0.981	0.985	0.974
	mean absolute error (MAE)	1.096	1.095	1.054	1.11	0.968	0.972	0.967
	root mean squared error (RMSE)	1.331	1.33	1.283	1.346	1.213	1.2	1.304

The first- and second-highest coefficients of determination in the models using images of both resolutions were for GBDT and random forests, respectively. These methods are based on decision trees and can create numerous conditional branches. The degree to which the fruit–camera distance affects the Mask RCNN output values depends on the size of the fruit to be captured (Fig 5A). A potential solution to overcome this issue while still accurately estimating fruit diameter is to separate the data based on the fruit–camera distance. An optimal regression model can then be created and applied to each distinct dataset. Since the decision tree uses conditional branching and can be applied to the aforementioned methods, the two decision tree–based methods were considered capable of estimating fruit diameter with higher accuracy. For instance, the GBDT model demonstrated the best results because of its applicability to the data. However, other regression methods lack the concept of conditional branching, and attempts to apply a single model to all data may have resulted in lower accuracy than decision tree–based methods.

**Fig 8:**
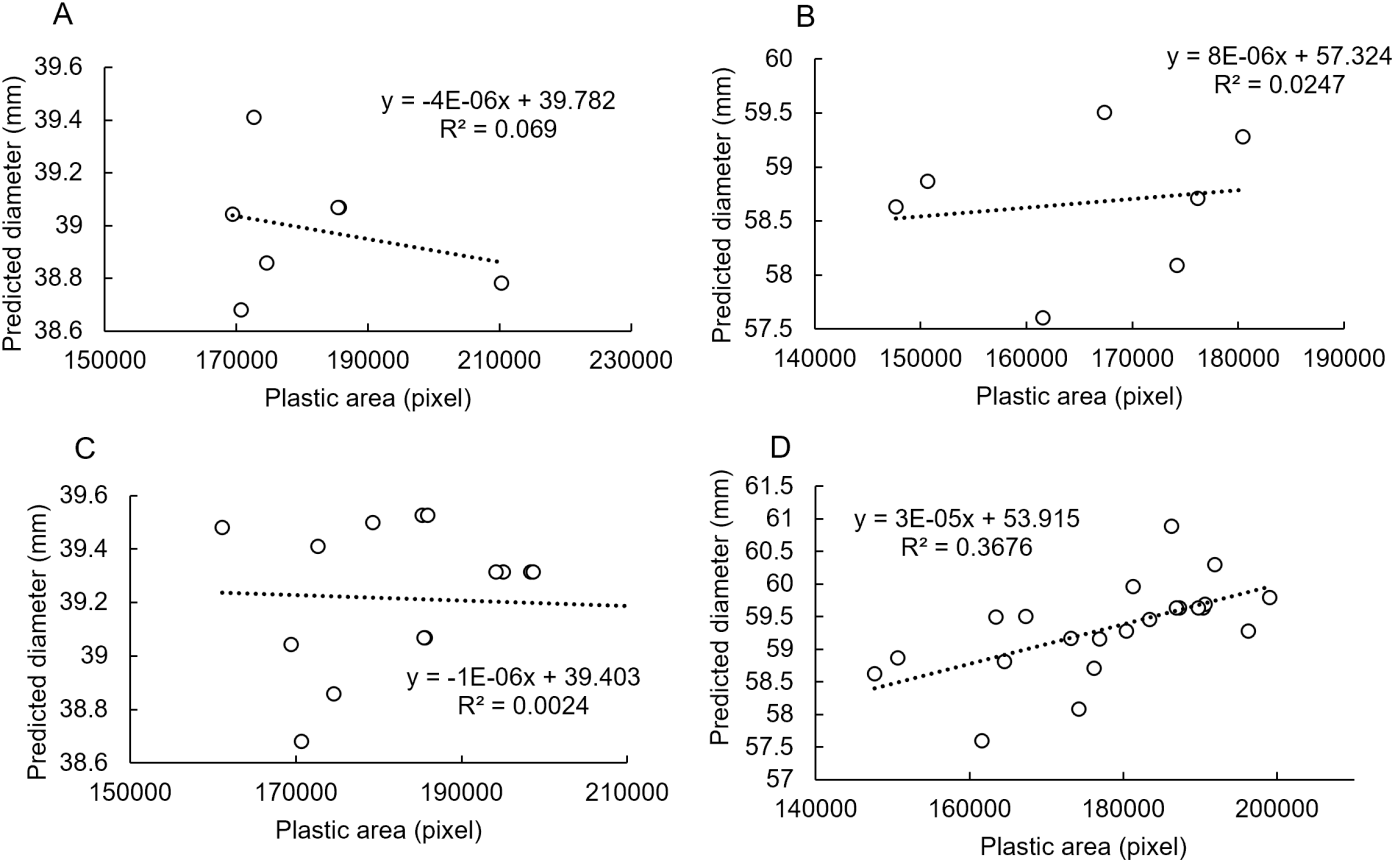
Relationship between predicted diameter by the GBDT model and plastic piece area. (A) 1 fruit 40 mm in diameter. (B) 1 fruit 60 mm in diameter. (C) 2 fruits 40 mm in diameter. (D) 3 fruits 60 mm in diameter. All data in the scatter plot are the same as those used in Fig. 2.

Fig 8 presents the distribution of the evaluated values by the GBDT model for multiple photos of the same fruit taken on the same date and multiple fruit images of the same size. In Figs 6A–6C, the area and area ratio of the plastic piece showed a strong negative correlation, while in Figs 8A–8C, no correlation was observed between the area of the plastic piece and the estimated fruit diameter. While a correlation was also not confirmed between the area of the plastic piece and the area ratio shown in Fig 6D, a positive correlation existed between the area of the plastic piece and the estimated fruit diameter (see Fig 8D). These results suggest that the GBDT model corrects for the bias caused by the camera–fruit distance in Figs 8A–C. The correction was even stronger in Fig 8D, resulting in a positive correlation.

Table 4 lists the effects of differences in explanatory variables on GBDT estimation accuracy. Compared to the model with all explanatory variables, the model with two areas as explanatory variables showed decreased accuracy in all evaluation functions, while the two models with either area and its ratio as explanatory variables showed equal or improved values in the evaluation functions. The two models using area ratios were almost equally accurate, but the model using fruit area and area ratio had the best value.

**Table 4:** Effect of Differences in Explanatory Variables on GBDT Prediction Accuracy.

	Explanatory variable	fruit+plastic+ratio	fruit+plastic	fruit+ratio	plastic+ratio
high resolution	coefficient of determination (R^2^)	0.988	0.986	0.988	0.988
	mean absolute error (MAE)	0.857	0.972	0.831	0.84
	root mean squared error (RMSE)	1.045	1.198	1.017	1.027
low resolution	coefficient of determination (R^2^)	0.985	0.981	0.986	0.985
	mean absolute error (MAE)	1.974	1.12	0.931	0.953
	root mean squared error (RMSE)	1.2	1.494	1.144	1.175

The relationship between the area of the plastic piece and the predicted fruit diameter in all models with only two explanatory variables did not change in trend from the model using all explanatory variables, suggesting that all models corrected for bias arising from the camera–fruit distance.

In the GBDT for the two explanatory variables, the models that showed high scores were those that used either the area or area ratio. However, models that used each independent value of area as an explanatory variable did not show high values, suggesting that the use of values indicating the relationship between the two areas as explanatory variables was effective in improving GBDT model accuracy in this study.

### Measurement of fruit enlargement by each method and its relationship to water stress

**Fig 9:**
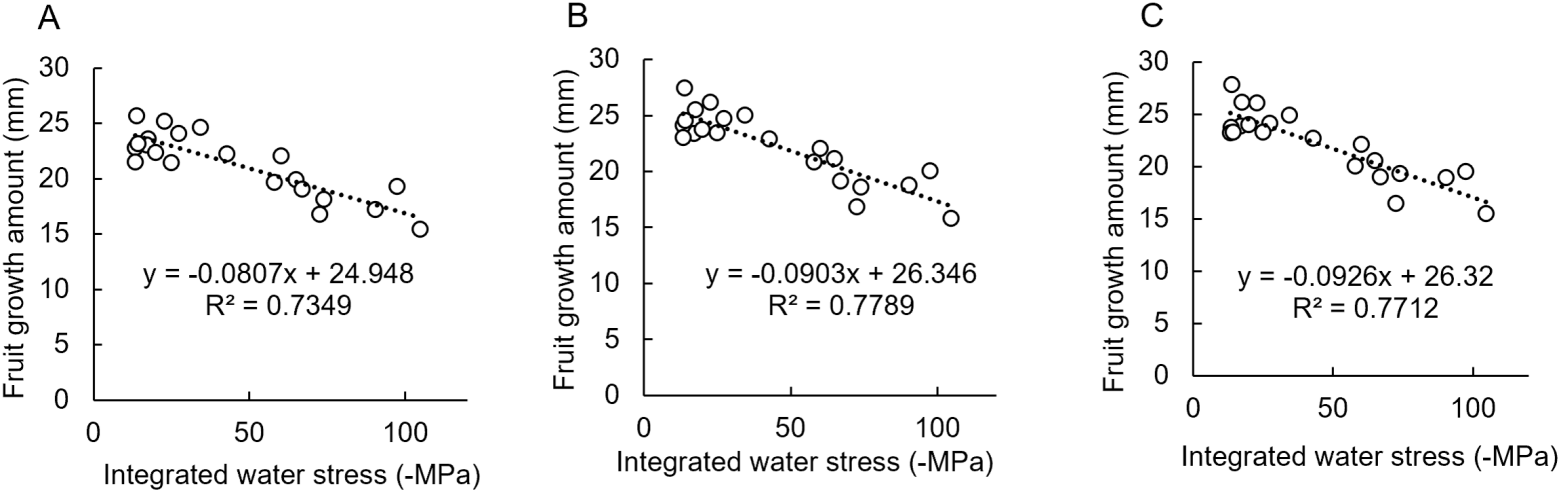
Relationship between fruit growth by each measurement method and integrated water stress. **(A)** Using a caliper. **(B)** Image analysis method for high-quality images. **(C)** Image analysis method for low-quality images. All figures are for the 2023 observation period (July 21–November 9).

Fig 9 shows the relationship between the amount of fruit growth calculated by the measurement method and SΨ_x_ during the entire measurement period. The developed method used the GBDT model, which uses the area and area ratio of the fruit with the highest score in the previous section. All measurement methods showed high correlations, and the developed method had a higher coefficient of determination than the caliper for both low-quality and high-quality images.

Table 5 lists the relationship between approximate monthly fruit growth and SΨ_x_. All measurement methods showed correlations except for the period from July 31 to August 26. A more detailed analysis of this interval revealed strong correlations in calipers and weak correlations in the developed methods for July 31–August 8, prior to the typhoon, whereas no correlations were found for all measurement methods for August 8–18 and August 18–26 (S1 Table). The August 8 fruit image was taken after rainfall, so some of the fruits had water droplets on them. When water droplets are attached to the contour of the fruit, the area in the image appears to be the same color as the fruit. Consequently, the developed method evaluated the size of some fruits as larger than they actually were, which is thought to have reduced the coefficient of determination.

**Table 5:** Relationship Between Fruit Growth and Integrated Water Stress at Different Times.

Methods used	Jul 21–Jul 31	Jul 31–Aug 26	Aug 26–Sep 28	Sep 28–Oct 25	Oct 25–Nov 9
caliper	0.6781	0.1203	0.5613	0.5352	0.3806
developed method (high-resolution picture)	0.6166	0.0008	0.4752	0.6327	0.7197
developed method (low-resolution picture)	0.506	0.0039	0.4179	0.6561	0.6467

All data represent coefficients of determination between fruit growth and accumulated water stress for each measurement method by time of year.

We observed that the typhoon flooded the field on August 16 and subsequently reduced the water potential of all trees. This result suggests that flooding causes water to enter the root zone. Maotani and Machida reported that fruits during or after drought stress were rapidly enlarged by irrigation and rainfall [28]. Iwasaki studied the correlation between fruit development after October and irrigated water stress in 2 years of data, reporting that a correlation was observed in years with dry soil in late October, while no correlation was found in years with wet soil during the same period [8]. Thus, the inflow of water into the soil markedly affected fruit growth, justifying the lack of correlation in August observed in this study.

Water potential, used as a measure of water stress, indicates how much pressure a leaf releases water from its petiole. It can also reflect how much force the leaf can exert to absorb water. When a large amount of water flows in at once, trees with higher water potential have a stronger ability to absorb water and thus absorb a larger amount of water, which is expected to result in a greater amount of fruit enlargement compared to trees with lower water potential. The time intervals of August 8–18 and August 18–26 were considered uncorrelated due to the mixed effects of rainwater inflow and normal water stress on fruit growth.

Looking at the trend of the coefficient of determination by time of year, calipers scored well until the end of September, while the developed method scored higher from October onward. The calipers showed a high correlation when the fruit was small, suggesting that the coefficient of determination decreased as the fruit grew larger. The equatorial plane of the fruit is a perfect circle during the juvenile stage but becomes less perfect as the fruit grows. A caliper can measure the lateral diameter, but the value is fragmented; obtaining an average value of the snarled circle is challenging. In contrast, the developed method estimates the lateral diameter based on two-dimensional data in the form of images, and the value is considered close to the fruit’s average value. This adaptability to fruits with complex shapes allowed the developed method to exhibit a high coefficient of determination, even for fruits in the late stages of maturity.

These results indicate that the developed method produces a higher coefficient of determination than calipers over the entire measurement period by obtaining a high correlation with SΨ_x_ in the late growth phase. Notably, future studies will explore the accuracy of the developed methods during the fruit-growing period.

## Conclusion

We developed a method to estimate the fruit diameter of satsuma mandarin by combining instance segmentation and regression modeling. The mean absolute errors for the best conditions were 0.831 and 0.931 for high- and low-resolution images, respectively. Since the test data were taken in a year different from the training data, the method was considered adaptable under varying field conditions. The Mask RCNN has also produced good detection rates in other studies. The absence of significant outliers in this study, when plotting the measured fruit diameter against the ratio of fruit to plastic piece area, suggests that both the fruit and plastic pieces were robustly detected. Consequently, the data were accurately input into a regression model to estimate fruit diameter with high precision.

A correlation was also observed between the results of the calculation of fruit size using this method and SΨ_x_, suggesting that this method can be used to estimate water stress conditions. However, the relationship between the developed method and water stress was higher in late maturity, while calipers showed a higher correlation in early maturity, suggesting that the optimal shooting conditions may differ depending on the time of year. Thus, further study is needed on the shooting conditions during the young fruit stage from July to September.

## Supporting information

S1 Fig Relationship Between Predicted Fruit Diameter by Area Ratio and Measured Fruit diameter. (A) using high resolution image (n = 9693). (B) using low resolution image (n = 9192).
(TIF)

S1 Data Sample dataset of high resolution pictures. Sample data sets on pixel values of fruit and plastic peace extracted from high-resolution images and measured fruit diameter.
(XLSX)

S2 Data Sample dataset of low resolution pictures. Sample data sets on pixel values of fruit and plastic peace extracted from low-resolution images and measured fruit diameter.
(XLSX)
