# Supplementary figures and images for: Development of a fruit size estimation method using Mask RCNN for water stress estimation in Satsuma mandarin trees
Source: PLoS One. 2025 Jul 11;20(7):e0324278. doi: 10.1371/journal.pone.0324278 (PMC12250200; doi:10.1371/journal.pone.0324278)

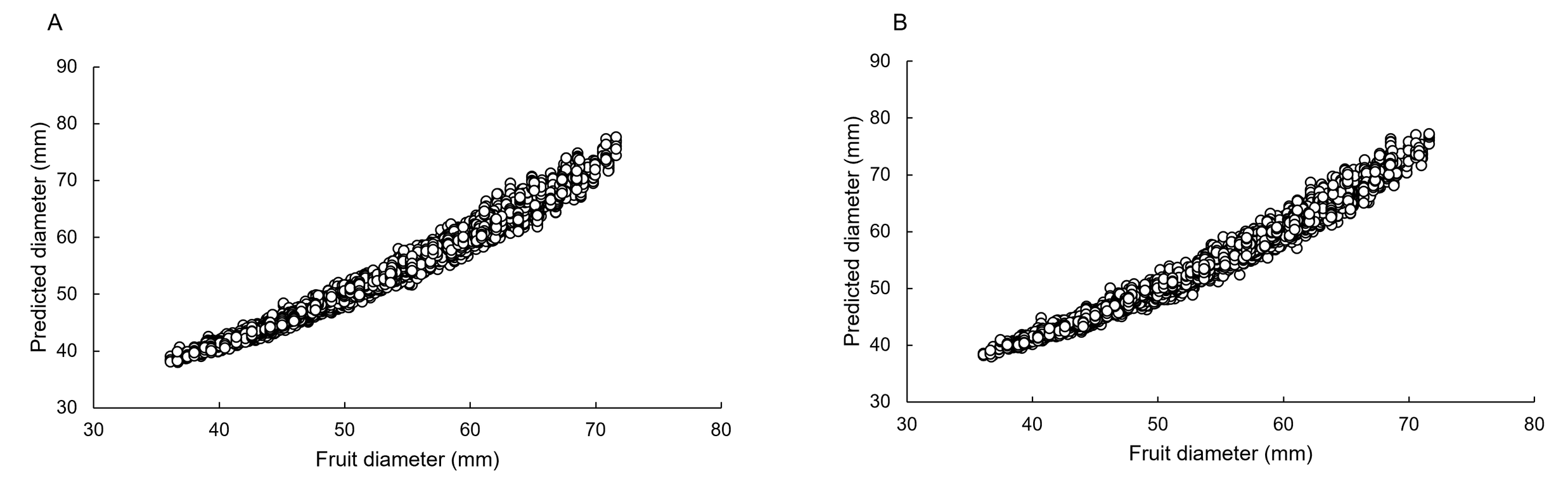

Supplement: S1 Fig — (A) using high resolution image (n = 9693). (B) using low resolution image (n = 9192). (TIF) [file pone.0324278.s001.tif]
